# A Genomic View of Alternative Splicing of Long Non-coding RNAs during Rice Seed Development Reveals Extensive Splicing and lncRNA Gene Families

**DOI:** 10.3389/fpls.2018.00115

**Published:** 2018-02-07

**Authors:** Edward A. Kiegle, Alex Garden, Elia Lacchini, Martin M. Kater

**Affiliations:** Department of Biosciences, University of Milan, Milan, Italy

**Keywords:** lncRNA, *Oryza sativa*, seed development, alternative splicing, RNA metabolism

## Abstract

Alternative splicing (AS) is a key modulator of development in many eukaryotic organisms. In plants, alternative splice forms of non-coding RNAs (ncRNAs) are known to modulate flowering time in Arabidopsis and fertility in rice. Here we demonstrate that alternative splicing of coding and long non-coding RNAs occurs during rice seed development by comparing AS in immature seeds vs. embryo and endosperm of mature seeds. Based on computational predictions of AS events determined from a Bayesian analysis of junction counts of RNA-seq datasets, differential splicing of protein-coding, and non-coding RNAs was determined. In contrast to roots, leaves, flowers, buds, and reproductive meristems, developing seeds had 5.8–57 times more predicted AS. Primers designed to span introns and exons were used to detect AS events predicted by rMATs in cDNA derived from early (milk) seed, embryo, and endosperm. Comparing milk seed vs. mature embryo and endosperm, AS of *MORC7* (a gene implicated in epigenetic gene silencing), was markedly different. Long non-coding RNAs (lncRNAs) also underwent AS during the transition from milk seed to mature embryo and endosperm, with a complex gene structure, and were more extensively processed than predicted by current genome annotation. Exon retention of lncRNAs was enhanced in embryos. Searching all 5,515 lncRNAs in the NCBI genome annotation uncovered gene families based on highly conserved regions shared by groups of 3–35 lncRNAs. The homologies to other lncRNAs, as well as homologies to coding sequences, and the genomic context of lncRNAs provide inroads for functional analysis of multi-exonic lncRNAs that can be extensively processed during seed development.

## Introduction

Alternative splicing (AS) is a fundamental mechanism for the functional diversification of the eukaryotic transcriptome. As more forms and functions of RNA are discovered, the importance of AS is being revisited as a means of not only creating multiple protein isoforms, but of producing and regulating short and long non-coding RNAs (Ulitsky, 2016). In plants, splicing is known to be involved in determination of flowering time, response to stress, circadian rhythms, and changes in temperature (Qüesta et al., 2016; Simpson et al., 2016; Ling et al., 2017; Verhage et al., 2017). Here we investigate the nature of alternative splicing of lncRNAs during rice seed development.

The breadth of RNA-seq data available provide a powerful resource to analyze AS. Myriad algorithms are available to determine the abundance of different isoforms present in RNA-seq output, but not all are capable of or excel at distinguishing AS between different conditions or developmental stages (Liu et al., 2014). However, rMATs allows pairwise comparison and is able to process RNA-seq data executed in replicates (Shen et al., 2014). While most programs are aimed at analysis of mammalian splicing, rMATS was shown to work well with data derived from plant experiments (Liu et al., 2014). Using this tool, it is possible so analyze junctions found in RNA-seq reads and produce a comparative analysis of AS variants between two conditions, treatments, or developmental stages.

Studies of AS during plant development revealed an important role for AS during flower development, and reproductive phase transition. The key role of AS in photomorphogenesis was elucidated in Arabidopsis, in which the splicing factor SPFS was found to directly interact with the photoreceptor system (Xin et al., 2017). Mutants disrupted in *SPFS* showed diminished responses to light signals of various wavelengths, and showed reduced AS in response to light stimuli. For example, mutant plants exposed to a 3 h pulse of red light suffered a 40% loss of AS which correlated with photomorphogenic defects. Xin et al. (2017) provide functional context for the well-established role of AS in light-controlled circadian rhythms in plants (Simpson et al., 2016). The importance of AS during development was also demonstrated by a recent study that showed marked cell-type specific differences in AS among developing root cell types (Li et al., 2016). Analysis of 15 root cell types revealed that for coding genes, AS is more strongly tied to developmental changes than cell type identity, and found one transcription factor for which the AS had functional impact (Li et al., 2016).

Profound changes in AS during stress have been shown in a number of monocot species. Drought-induced AS affects markedly different gene sets in vegetative vs. reproductive tissues in maize, underscoring a tissue-dependent AS response to stress (Mei et al., 2017b). In wheat, drought and heat stress increase not only the overall number of genes undergoing AS (from 200 to 4,056), but also the percentage of AS genes with altered transcriptional levels (from 12 to 40%) (Liu et al., 2017). A study of a drought-resistant rice breeding line found drought-specific splicing events as well as differences in the functions of genes undergoing AS (Wei et al., 2017). Evidence for evolutionary conservation of AS events across syntenous species suggests that the functions of AS in the stress responses of wheat and maize are likely to exist in rice as well (Mei et al., 2017a).

While most studies have focused on protein coding genes, AS can also occur in the transcription of non-coding RNAs. Of particular interest is splicing of lncRNAs, which have been shown to regulate AS during *Arabidopsis* root development (Bardou et al., 2014). Recently, a genome-wide screen found a population of lncRNAs specifically expressed and functionally involved during all stages of rice reproductive development (Zhang et al., 2014). Two of the most extensively-studied examples of lncRNA function in plants as key developmental switches: COOLAIR/COLDAIR in Arabidopsis vernalization and LDMAR in photoperiod-sensitive male sterility in rice. In both cases, multiple splice forms of functional ncRNAs were discovered. Nonetheless, little is known about the frequency or complexity of lncRNA splicing in plants. In fact, there is the potential for feedback or feedforward effects, as it was recently shown that lncRNA can compete for splicing regulator binding sites to modulate AS during root development (Bardou et al., 2014).

lncRNAs in plants affect gene and protein expression in direct and indirect ways. Chromatin state and structure are the basis of the activities of *COLDAIR* on flowering time (histone modification), *HID1* on photomorphogenesis (intron binding), and *APOLO* on auxin action (chromatin looping). *COOLAIR* and *LDMAR*, for example, also function by modulating the epigenetic states of their target promoters (Ding et al., 2012; Csorba et al., 2014). Although lncRNAs antisense to coding transcripts are the most obvious, lncRNA functions are much more complex, and range from control of protein localization to “molecular sponges” (Wang and Chekanova, 2017).

Lastly, links have been established between AS and epigenetic control of gene expression. Reproductive development in plants involves multiple levels of epigenetic control of cell and organ identity. In rice, the germ cells are targets of complex methylation dynamics, and parent of origin effects are known to play a major role in endosperm development (Park et al., 2016). DNA methylation state is a key regulator of endosperm-specific genes such as those coding for storage proteins and starch biosynthesis enzymes (Zemach et al., 2010). Moreover, Koltunow et al. elucidated an example of parentally-determined alternative splicing and polyadenylation of an mRNA encoding a DUF-domain protein in developing rice seeds (Luo et al., 2011). We sought to investigate the magnitude of AS in developing rice seeds, particularly in light of possible involvement of long non-coding RNAs in the formation of the embryo and endosperm.

At the whole seedling level, plants with disrupted CG methyltransferase OsMet1-2 manifest abnormally high levels of AS compared to wild type plants (Wang et al., 2016). Only 19% of differentially expressed genes were in the pool of differentially spliced genes, suggesting independent regulation of AS via CG methylation. We re-analyzed the *OsMet1-2* mutant vs. wild type dataset to compare the mutant seedling AS to the AS in developing seeds using the same AS algorithm.

Global analysis of alternative splicing relies upon the nature and quality of the annotation of RNA species in the genome. Analysis of AS of lncRNAs is challenged further by the veracity of the annotation algorithm used to define coding and non-coding RNAs, and/or the criteria employed to interpret RNA-seq data for splice junctions. The NCBI annotation has the advantage of incorporating information from cDNA, EST, and RNA-seq databases to support computational predictions. Using lncRNA AS events elucidated with cDNA from developing seeds, the accuracy and precision of the annotation was testable. To this end, a sequence-based (rather than positional) approach was used to determine the nature of lncRNA gene families in the rice genome, and to explore homology of lncRNAs to protein coding sequences within and outside of the *Oryza* genus and the monocotyledon class.

## Methods

### RNA-seq data processing and analysis

Numerous datasets were obtained from the NCBI Sequence Read Archive (https://www.ncbi.nlm.nih.gov/sra) to test their suitability for alternative splicing analysis with rMATS. Detailed analysis was performed of datasets from the following studies: developmental time course GSE56463 (Wang et al., 2015), reproductive meristem series PRJNA306226 (Harrop et al., 2016), rice methylase mutant OsMet1-2 SRP043447–SRP043449 (Wang et al., 2016), and isolated rice reproductive cells GSE50777 (Anderson et al., 2013).

For rMATS analysis, a.gtf file was constructed from the NCBI Reference IRGSP-1.0 Primary Assembly: GCF_001433935.1_IRGSP-1.0_genomic.gff. Chromosome names were converted from NCBI to IRGSP convention, and consequently the Cufflinks gffread tool was used to convert the.gff file to a.gtf file. STAR was used to generate genome files from the IRGSP FASTA genome sequences (ftp://igenome:G3nom3s4u@ussd-1.0/1.0.tar.gz) and the above mentioned.gtf file. RNA-seq experiment FASTQ files (including replicates when available) were obtained with the SRAtoolkit fastq-dump command.

Gene descriptions were obtained with the NCBI batch interface. Gene Ontology analysis was performed using loci that met significance cutoffs (*p* < 0.005 and FDR < 0.05) using PANTHER's statistical overrepresentation test with the default PANTHER GO-slim Biological Process annotation and Bonferroni correction settings (Mi et al., 2017). rMATs output and gene descriptions can be found in Supplementary Data Sheets 1, 2.

### Plant growth and sample isolation

*Oryza sativa* var. Nipponbare was grown in growth chambers at 28°C with 16 h light (long day) for 8 weeks followed by growth 12 h light (short day) to induce flowering. Milk seeds were harvested 8–9 days post-pollination, and mature seeds were harvested 7 days later. Embryos were manually separated from endosperm with a razor. Samples were collected in triplicate. RNA was isolated from whole milk seed and isolated embryo and endosperm using a modified TriZol method (Wang et al., 2012) lacking a second organic extraction but with DNaseI treatment, followed by LiCl precipitation and a second DNase treatment and precipitation before cDNA synthesis. cDNA was prepared using 2 μg of total RNA and Maxima reverse transcriptase (Thermo Fisher) in a 20 μl reaction volume.

### Semi-quantitative RT-PCR and amplicon analysis

A total volume of 10 μl included 1 μl of cDNA in a standard GoTaq polymerase (Promega) mix. PCR was performed for 25–30 cycles (95°C for 30 s, 57°C for 30 s, 72°C for 30 s). Primers flanking predicted splicing events were designed using PrimerSeq (Tokheim et al., 2014) when possible, or manually based on annotated exon/intron predictions. Primers for ubiquitin (Ding et al., 2012) (LOC4332169) and elongation factor 1 (LOC4331812) were used to ensure consistent concentration determination, and sub-saturating PCR conditions, and the absence of gDNA contamination. Amplicons were cut and purified from 1.5 or 2% agarose gels (kit), subcloned into the pGEM T-easy vector and sequenced. Alignments were performed with the T-Coffee alignment package (Notredame et al., 2000).

### Analysis of lncRNA genes and families

A custom program, available at GitHub (https://github.com/agardb/NCBI-RGA) first called a genome assembly from the NCBI (ftp://ftp.ncbi.nlm.nih.gov/genomes/all/GCF/001/433/935/GCF_001433935.1_IRGSP-1.0/GCF_001433935.1_IRGSP-1.0_rna.gbff.gz), then searched for all features annotated as “ncRNA” and tabulated their locus ID, length, and number of exons. When run with rMATS output, it rejected all LOCs not found in one of the rMATS output files with *p* < 0.005 and FDR < 0.05. When run without rMATS output, it either filtered out all but the longest or all but the shortest isoforms. It then collected BLASTN results from the NCBI BLAST tool searching the refseq_RNA database with the longest lncRNA isoform. Consequently, alignments to ribosomal RNA were filtered out, and the results split into matches to *O. sativa* ncRNA, *O. sativa* protein-coding RNA, or alignments to RNA from organisms other than *O. sativa*. The NCBI nucl_gb.accession2taxid table was used to append the species name of the match to the non-species file. Lastly, for determination of lncRNA families, results were filtered by length (≥100 bp significant match length) and screened against the Type I transposon dataset downloaded from the RiTE *Oryza* transposable elements database to remove spurious ncRNA families associated with TE matches (Copetti et al., 2015). Gene family data are available in Supplementary Data Sheet 3.

## Results

Preliminary analysis of reproductive meristem RNA-seq data clusters (Harrop et al., 2016) revealed putative RNA binding proteins/splicing factors. To elucidate the potential importance of alternative splicing in seed development, we chose to analyze RNA-seq datasets with rMATS. A comprehensive analysis of several methods (rDiff, DiffSplice, rMATS, CuffDiff, and DesSeq) designed to identify alternative splicing in RNA-seq datasets identified almost no convergence in the overall predictions (Liu et al., 2014). However, the rMATS algorithm stood out as the most robust tool to identify AS events when tested with “real” datasets of validated biological events, particularly when genome annotation is robust, and splicing events are simple. NCBI *O. sativa* japonica group Annotation Release 101 integrates extensive RNA-seq data to support intron and exon calls, as well as data from EST, GenBank, and full length cDNA datasets. 38,325 RNAs of 40,907 (93.7%) are considered “fully supported” (https://www.ncbi.nlm.nih.gov/genome/annotation_euk/Oryza_sativa_Japonica_Group/101/).

**Figure 1:**
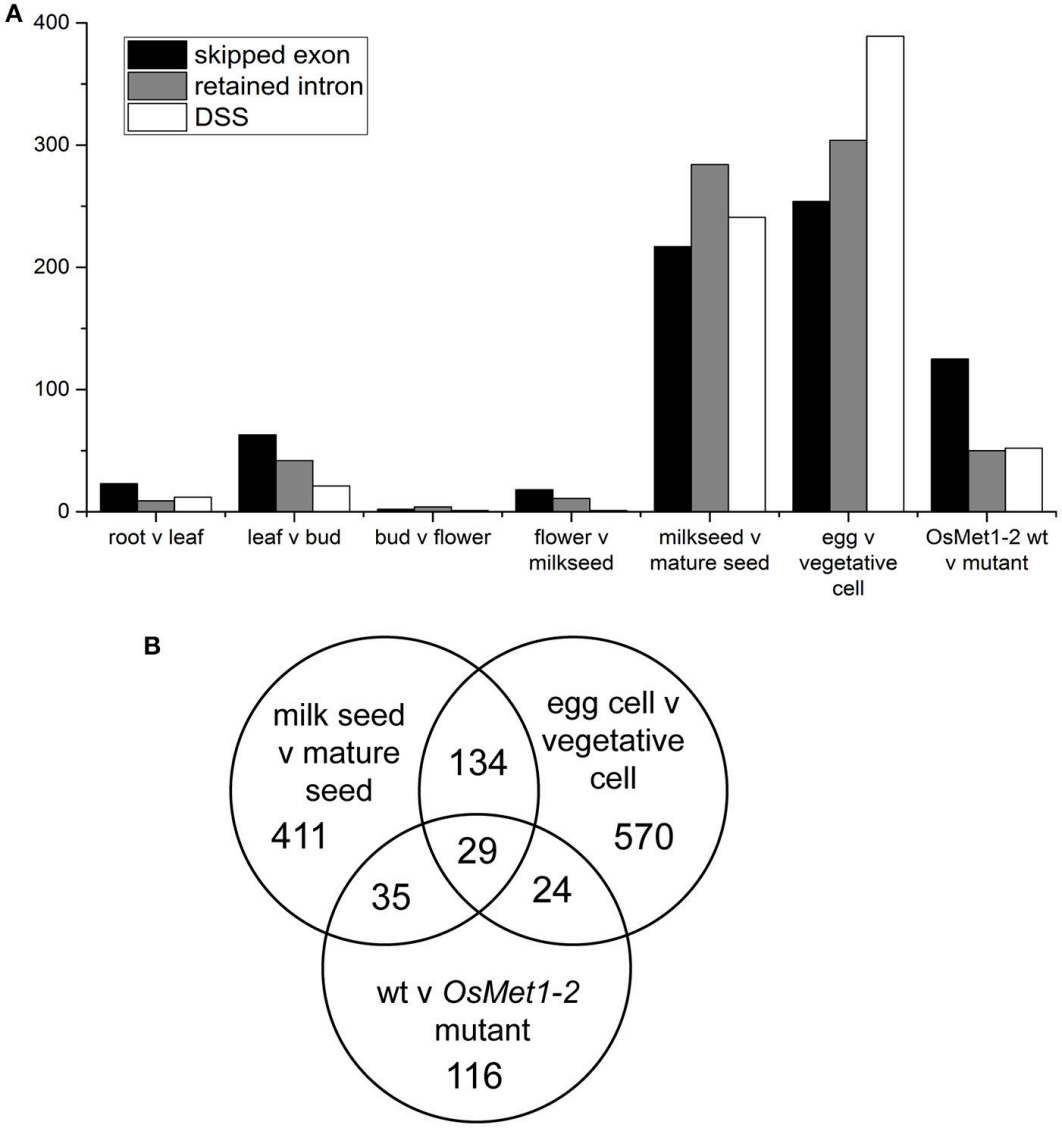
**(A)** Alternative splicing events identified in comparisons of tissues, gametes (egg and vegetative cells), and seedlings of lethal methylase mutant *OsMet1-2*. Numbers represent statistically significant alternative splicing events called by rMATS at *p* < 0.005 and FDR of < 0.05. **(B)** Venn diagram of predicted splicing events shared by and unique to the three datasets with significant AS.

Therefore, rMATS was used with the current NCBI build to screen the rice meristem dataset as well as numerous publicly available RNA-seq datasets to quantify the relative number of statistically significant skipped exon (SE), retained intron (RI), and differential 5′ or 3′ splice site (DSS) events. Surprisingly, relatively few events were detected in reproductive meristems (13 events) and a full series of plant tissues, 44 in root vs. leaf, 126 in leaf vs. bud, from 7 in bud vs. flower and 30 in flower vs. early seed (milky endosperm, milk seed) (Wang et al., 2015). However, comparison of milk seed vs. mature seed identified 742 events (Figure 1A; Supplementary Data Sheets 1, 2). The highest numbers of events (947) were found in egg vs. vegetative cell comparisons (Anderson et al., 2013). Egg cell vs. sperm cells showed much lower AS (173 events). Interestingly, re-analysis of RNA-seq data from seedlings of wild type and (seedling lethal) methylase mutant *OsMet1-2* (Wang et al., 2016) scored a high level of skipped exons, and to a lesser extent, RI and DSS events (Figure 1A).

In order to detect trends in gene function among alternatively spliced genes, gene ontology analysis was performed on the three datasets with the highest levels of AS (Table 1). In both milkseed vs. mature seed and egg cell vs. vegetative cell datasets, regulation of phosphate metabolism was elevated. This enhancement (9.69- and 11.5-fold, respectively) is most likely tied to regulation of phosphorylation. More interestingly, the milk seed vs. mature seed AS events were conspicuously enriched in RNA metabolism itself, including RNA splicing via transesterification and RNA splicing via spliceosome (7.68 and 7.33-fold) as well as transcription from the RNA polymerase II promoter (4.34-fold). mRNA splicing via spliceosome was present in egg cell vs. vegetative cell to a lesser extent (5.59-fold) and transcription from RNA Pol II promoter to a similar extent (4.86-fold). Remarkably, two of only three GO categories enriched in the comparison of wild type seedlings vs. *OsMet1-2* mutant seedlings were RNA processing via transesterification (10.59-fold) and via spliceosome (10.10-fold). These data hinted at interactions between AS of components of the splicing machinery and processes involved in methylation or chromatin modification (Reddy et al., 2012). Alternative splicing of transcripts coding for components of the splicing machinery has been observed in plants, but the relationship of this to chromatin modification remains unclear (Reddy, 2007; Gulledge et al., 2012).

**Table 1:** Gene Ontology statistical enrichment based on predicted alternative splicing events from developing seeds, reproductive cell types, and *OsMet1-2* mutant seedlings with PANTHER overrepresentation test (*p* ≤ 0.05).

**Milkseed vs. Mature Seed**	**Fold enrichment**	**Genome**	**Input**	**Expected**	***P*-value**
Regulation of phosphate metabolic process	9.69	107	6	0.62	8.08E-03
RNA splicing, via transesterification reactions	7.68	225	10	1.3	1.96E-04
mRNA splicing, via spliceosome	7.33	283	12	1.64	2.72E-05
Transcription from RNA polymerase II promoter	4.34	877	22	5.07	2.89E-06
Intracellular protein transport	3.71	1491	32	8.63	6.89E-08
Organelle organization	3.36	669	13	3.87	3.27E-02
Signal transduction	2.94	999	17	5.78	1.74E-02
Nitrogen compound metabolic process	2.88	2582	43	14.94	1.48E-07
Biosynthetic process	2.26	2293	30	13.27	6.61E-03
Protein metabolic process	1.90	3552	39	20.55	2.04E-02
**EGG CELL vs. VEGETATIVE CELL**					
MAPK cascade	12.3	100	7	0.57	4.02E-04
Regulation of phosphate metabolic process	11.5	107	7	0.61	6.25E-04
Chromatin organization	7.95	199	9	1.14	5.13E-04
DNA metabolic process	6.93	507	20	2.89	5.17E-09
Protein localization	5.61	282	9	1.61	7.85E-03
mRNA splicing via spliceosome	5.59	283	9	1.62	8.06E-03
Transcription from RNA polymerase II promoter	4.86	578	16	3.3	6.04E-05
Intracellular protein transport	4.12	1491	35	8.51	5.52E-10
Response to stress	3.47	1012	20	5.78	3.92E-04
Nitrogen compound metabolic process	3.33	2582	49	14.74	4.75E-11
Cellular protein modification process	2.53	1666	24	9.51	7.33E-03
**WILD TYPE vs. *OSMETL*-2 MUTANT (SEEDLING)**					
RNA splicing, via transesterification reactions	10.59	225	10	1.3	2.25E-02
mRNA splicing, via spliceosome	10.10	283	12	1.64	6.05E-03
Cellular process	1.84	9051	112	52.37	3.53E-02

*“Genome” indicates the number of that type of gene across the entire genome, while “Input” indicates the number of that type of gene in the experimental dataset*.

To further investigate these trends, a Venn diagram was generated to examine gene enrichment common to and unique to the three data sets (Figure 1B). The 29 AS loci shared by seeds, gametes, and the methylase mutant were enriched 34.8-fold for mRNA processing. Loci that are involved in RNA processing that are themselves targets of AS included regulator of nonsense transcripts homolog (LOC4343292), Ser/Arg-rich splicing factor RSZ21 (LOC4330968), Luc7-like RNA binding protein (LOC4334750), and histone-arginine methyltransferase CARM1 (LOC4344248). Alternatively spliced loci unique to the wild type vs. methylase mutant seedling showed no significant enrichment categories, consistent with a lethal deregulation of splicing. In contrast, loci unique to the milk seed vs. mature seed comparison were primarily enriched in transcription from the RNA polymerase II promoter (3.95-fold) and intracellular protein transport (3.65-fold). The gamete comparison was enriched 14.46-fold in MAPK cascade genes, 11.26-fold in phosphate metabolism, 7.27-fold in chromatin organization, and 6.41-fold in DNA repair.

We focused on alternatively spliced loci enriched in RNA processing category of the GO analysis, as well as alternatively spliced long non-coding RNAs (lncRNAs) that may play a role in seed development. The rMATS analysis of the milkseed vs. mature seed RNA-seq experiment (Figure 1A) identified 217 possible skipped exon (SE) events, 247 possible retained intron (RI) events, 85 potential alternative 5′ splice sites (A5SS), and 131 alternative 3′ splice sites (A3SS). Mature seed contains embryo and endosperm, highly differentiated and biologically distinct tissues. Therefore, embryo and endosperm of mature seeds were manually separated and analyzed independently. The GO enriched skipped exon set was used to identify the AS events in the milk seed and mature embryo and endosperm.

**Figure 2:**
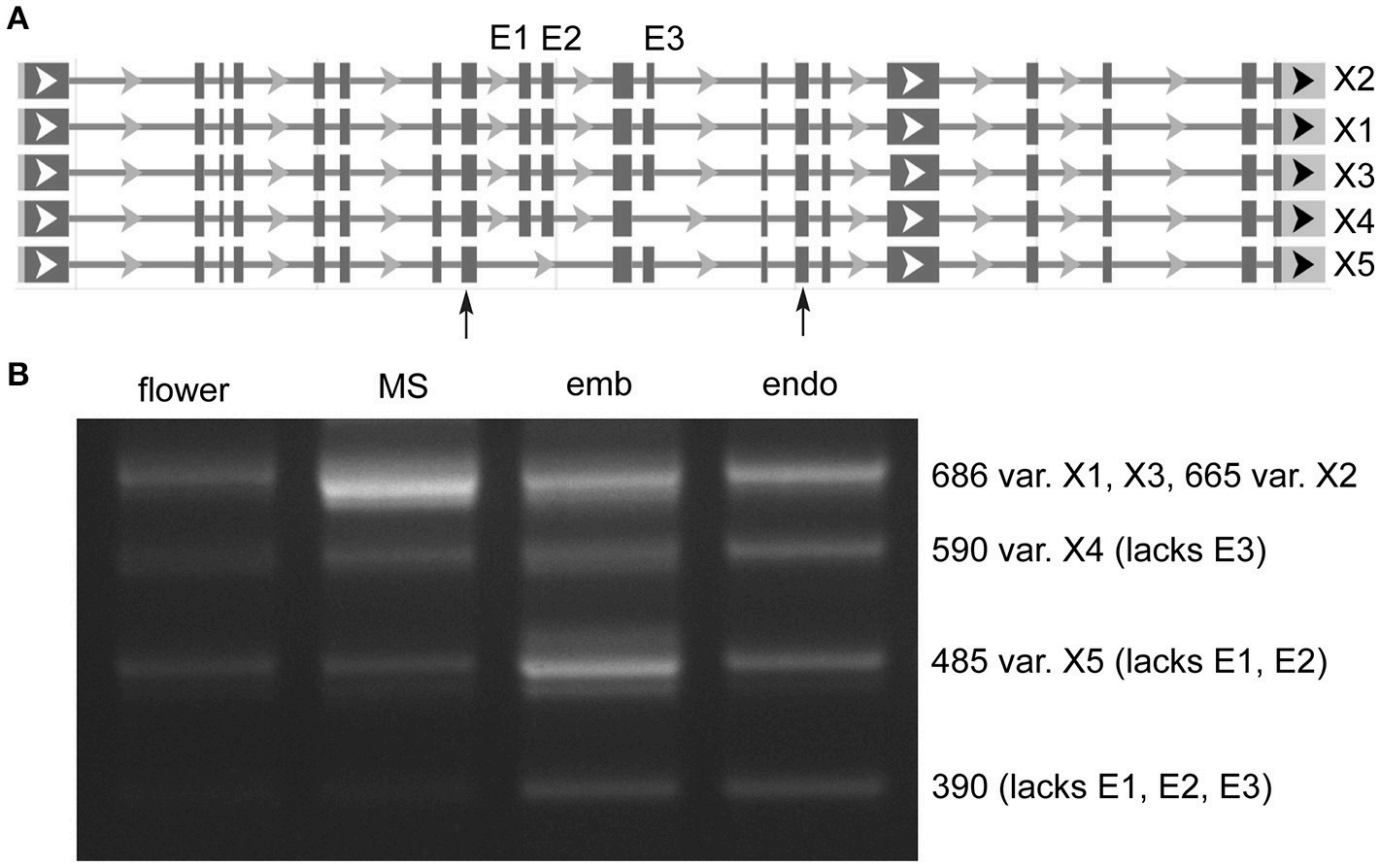
Alternative splicing of MORC7 (LOC4329338) skipped exons in milk seed, embryo, and endosperm. **(A)** Gene structure. Primers flanking skipped exons are indicated by arrows. **(B)** RT-PCR analysis of splice variants (MS, milk seed; emb, embryo; endo, endosperm). Three exons are predicted. An unannotated variant lacking all three exons is also present in developing seeds.

Distinct AS of a protein coding gene was observed in *MORC7* (Figure 2), for which five variants are annotated. Very low expression was seen in flower, while milk seed expression was predominated by the largest variant that contained all three exons (X1). In embryo, X1 expression was reduced, and expression of a variant (X5) lacking two exons was increased. In addition, a variant absent in the annotation lacking all three exons was present at low levels only in embryo and endosperm. The absence of exons in the shorter variants is predicted to alter the ATPase domain of the resulting protein (discussed below).

Given the roles of non-coding RNAs in rice reproduction (Komiya et al., 2014) and imprinting during seed development (Rodrigues et al., 2013), we were interested in assessment of the extent of lncRNA AS predicted by rMATS in milk seed and mature embryo and endosperm. lncRNAs were identified in the rMATS output: 17 SE, 55 RI, 11 alternative 5′ SS, and 23 alternative 3′ SS. While the ability to design high quality primers in restricted regions, as well as the large size of most introns limited the scope of validation, it was possible to assess 23 lncRNAs for alternative splicing using RT-PCR for both skipped exons and retained introns. Rejecting any that showed similar splice forms in different tissues, 15 lncRNAs with discernible AS in milk seed, embryo and/or endosperm were identified. Of these, 10 lncRNAs with the most distinct AS patterns were further analyzed.

**Figure 3:**
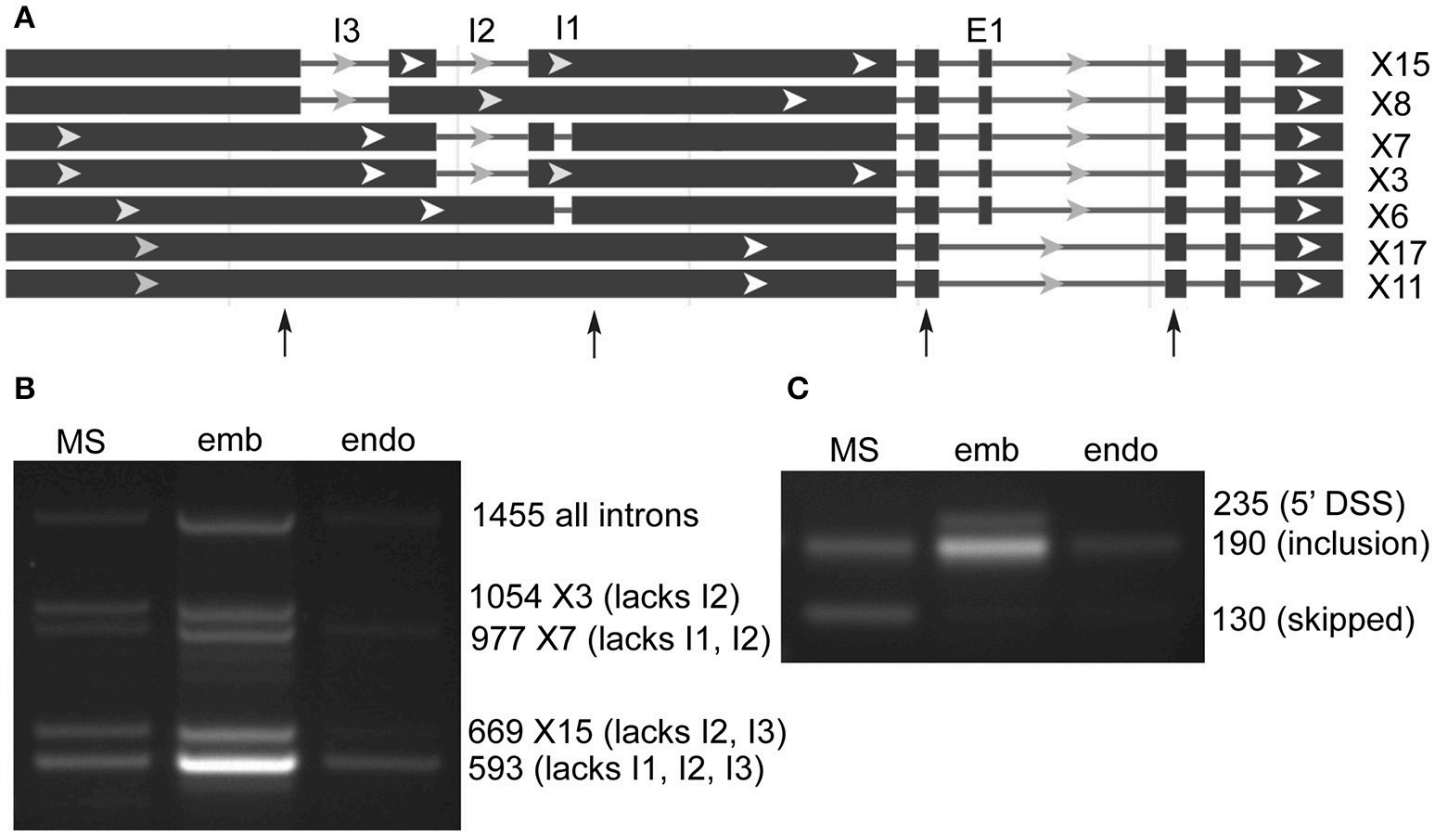
Alternative splicing of ncRNA LOC9270896 skipped exons and retained introns in milk seed, embryo, and endosperm. **(A)** Gene structure. Primers flanking skipped exons and retained introns are indicated by arrows. **(B)** RT-PCR analysis of retained intron amplicons (MS, milk seed; emb, embryo; endo, endosperm). The 1,455 bp band containing all three introns is present in 15 of 20 predicted variants, whereas the 593 bp band from which all introns are spliced out is not predicted but predominates in embryo. **(C)** Exon skipping is seen in milk seed. In embryo exon retention and an unannotated differential 5′ splice site variant are present.

lncRNA LOC9270896 was found in both SE and RI rMATS AS prediction sets, and has a complex structure with 20 predicted RNA sequence variants (Figure 3A). The genomic region on chromosome 6 is highly enriched in genes for transporters (glutamate receptor-like 2.5, 2.7, 2.8, 2.9-like; UDP sugar transporter, cyclic nucleotide gated channel 17). Analysis of the predicted intron and exon splicing sites showed a strong enrichment of (unannotated) intronless transcript in embryo, with low levels of larger variants in milk seed, and in endosperm only the smallest, intronless variant was observed (Figure 3B). In contrast, the skipped exon was retained to a large extent in embryo, as well as an unannotated alternative 5′ splice site variant. The exon was skipped in milkseed and only very low levels of an inclusion product were detected in endosperm (Figure 3C). Three regions (605, 382, 208 bp) of lncRNA LOC9270896 were highly conserved (>95% identity) in lncRNAs on chromosomes 11 (LOC9269643) and 12 (LOC9268659).

**Figure 4:**
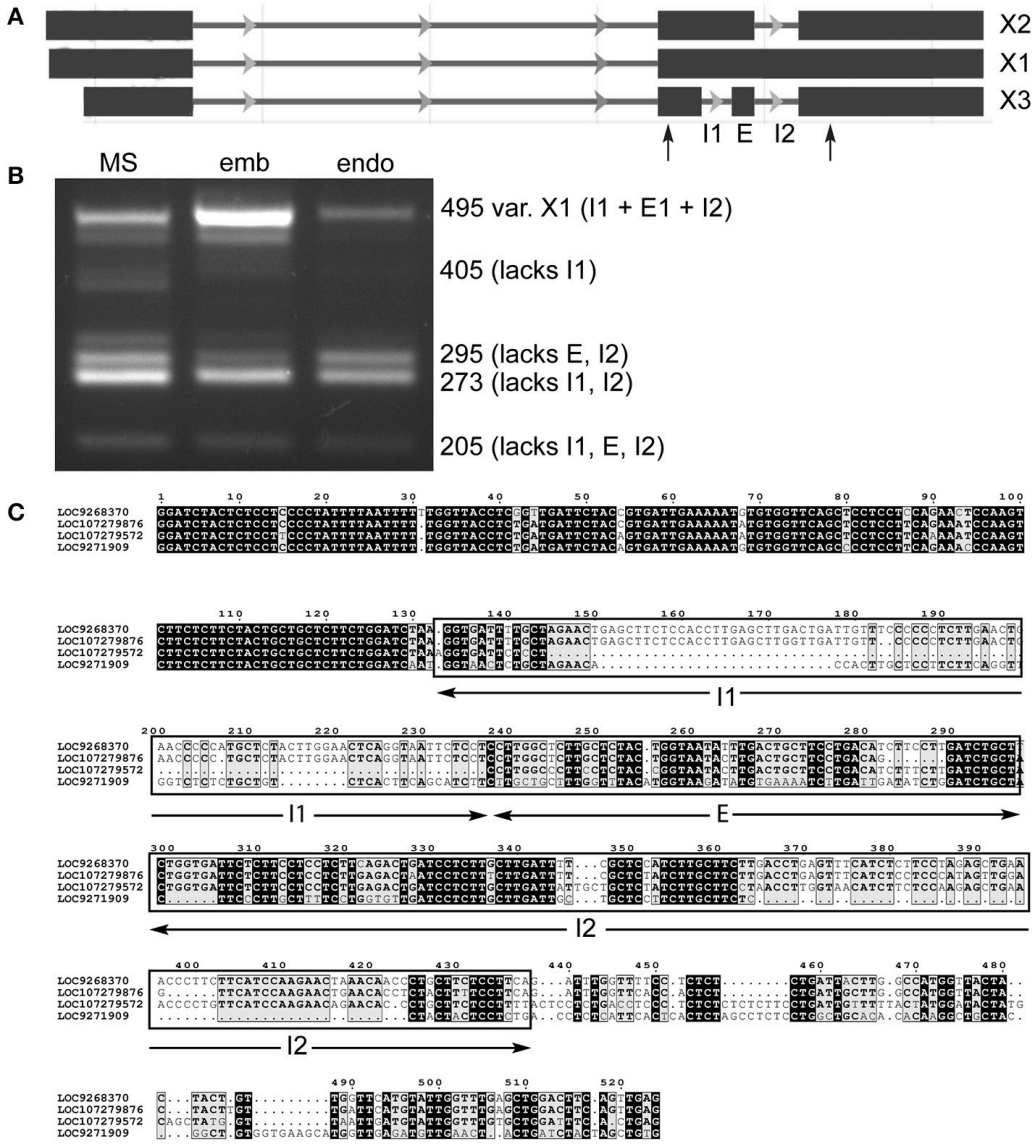
Alternative splicing of lncRNA LOC107279876 retained introns in milk seed, embryo, and endosperm. **(A)** Gene structure. Primers flanking retained introns and skipped exon are indicated by arrows. **(B)** PCR amplicons containing different splice variants (MS, milk seed; emb, embryo; endo, endosperm). **(C)** alignment of conserved ncRNA sequences on chromosomes 2, 1, 12, and 8, respectively.

It was possible to find evidence for all three predicted variants of lncRNA LOC107279572 (Figure 4A), as well as an unannotated transcript that is more extensively spliced (Figure 4B). Specifically, smaller variants lacking two introns or an intron and exon were prevalent in milk seed, while embryo showed a strong presence of the “full length” transcript (X1), which was diminished in endosperm. In the process of sequencing the amplicons, we identified amplicons from another locus, LOC107279872. After searching for homologs, it was found that part of the lncRNA was highly conserved in loci on four different chromosomes (Figure 4C). Searching the conserved region against a database of published monocotyledonous miRNAs (Kozomara and Griffiths-Jones, 2014) identified a region homologous to seed-specific wheat miRNA miR9661, which was identified as a target of an F-box transcription factor (Han et al., 2014). The genomic context of LOC107279572 contains two uncharacterized protein coding genes, two lncRNAs, and an S-type anion channel (LOC4325432).

**Figure 5:**
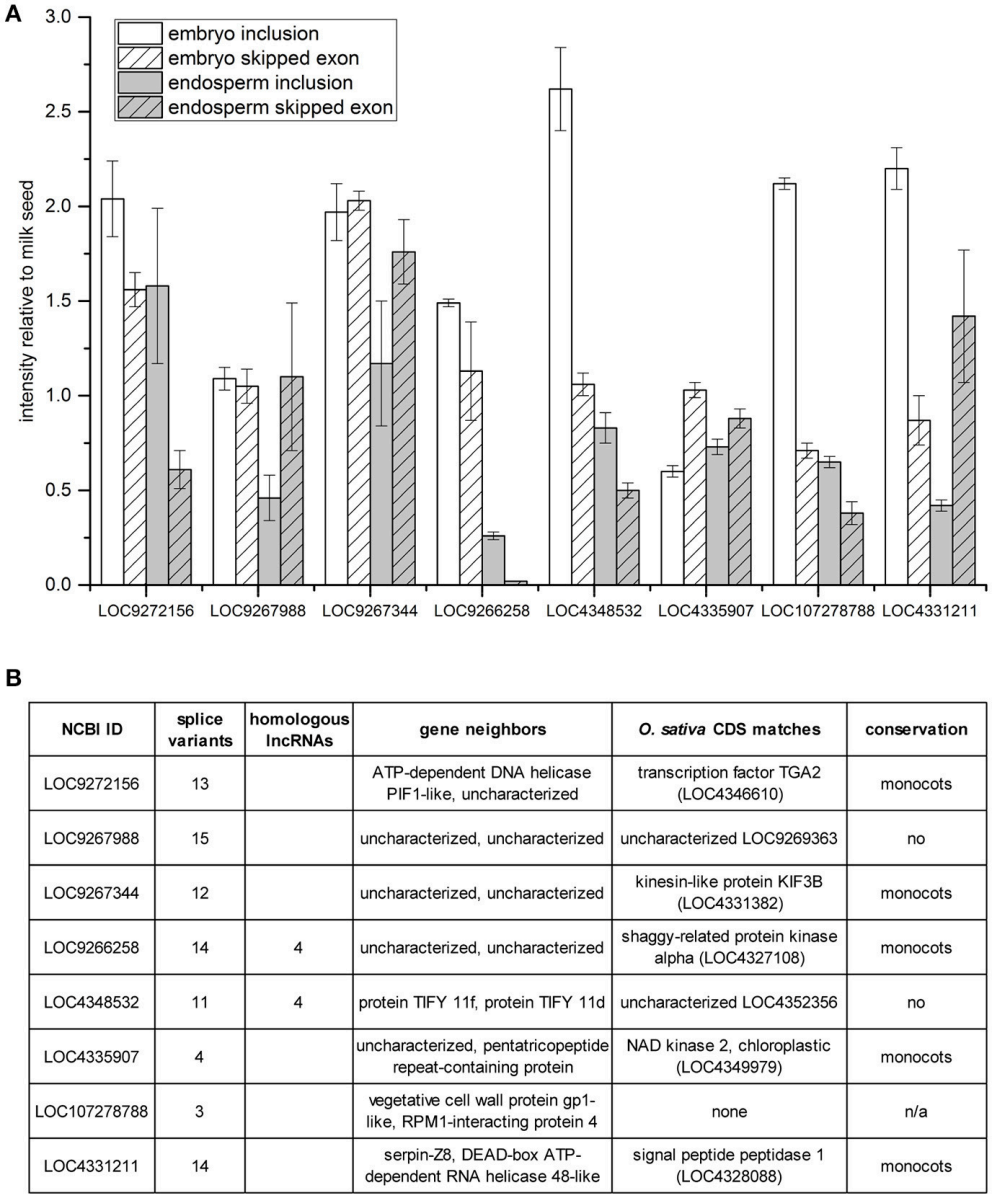
Exon skipping/inclusion of 8 lncRNAs in embryo and endosperm. **(A)** Band intensities relative to milk seed ± standard deviation (*n* = 3). **(B)** Homologs, genomic context, sequence matches, and evolutionary conservation of lncRNAs in **(A)**.

A survey of skipped exon AS of eight additional lncRNAs was undertaken with multiplex RT-PCR using primers designed to amplify products for both the inclusion and the skipped exon RNA variants (Figure 5). Band intensities present in embryo and endosperm were normalized to those measured in milk seed (Figure 5A). The predominant trend, in five cases, is inclusion of exons in embryo compared to milk seed. In endosperm, half the lncRNAs showed enhanced inclusion relative to milk seed while half showed the enhanced exon skipping relative to milk seed. Six of the eight lncRNAs had more than 10 predicted splice variants, and five were members of gene families. Sequence analysis determined that five of the eight lncRNAs (as well as LOC107279876, see above) were conserved in one or more monocotyledonous plants with reference genomes at NCBI (Figure 5B).

The potential exists for “non-coding” RNAs to have at least some forms that may express short peptides. To examine this, every splice variant of the 59 predicted lncRNAs with rMATS-predicted AS in milk seed vs. mature seed were analyzed with CPC2 (Kang et al., 2017), and those with putative coding potential (25%) were manually screened to see if the skipped exon or retained intron event(s) correlated to coding potential. Predicted peptides ranged from 105 to 236 amino acids. For three RI candidates there was a discrepancy between the NCBI annotation as noncoding and the CPC2 prediction as coding for all variants. Two of these were supported by cDNA evidence as protein coding. Therefore, it is likely that the NCBI annotation as non-coding is incorrect.

There were 6 cases of rMATs skipped exon events in loci with CPC2-predicted coding potential. Three of these were validated with PCR. In two cases (LOC9267344, 12 sequence variants, 4 potentially coding; LOC9272156, 13 sequence variants, 3 potentially coding) the coding potential was not affected by the presence or absence of the exon. However, in the case of LOC9270986 (20 variants, 1 predicted to code 129 amino acids), the coding potential requires the absence of an intron, the presence of another, and the presence of an exon (Figure 3). The expected 1,066 bp band for the coding variant was not detected, but notably embryo had suppression of introns and inclusion of the necessary exon. Thus, control of splicing could regulate the expression of the peptide. In fact, of the 20 splice variants, the 2 with skipped exons, and therefore no coding potential, were only observed in milk seed (Figure 3).

In order to determine if any of the ncRNAs analyzed were spliceosomal, snoRNA, miRNA precursors, or other known RNA forms, the SE and RI ncRNAs from the seed data set were analyzed with rFAM, which contains 178 RNA families for rice (Kalvari et al., 2018). The search yielded three results, all matching miR1846, a microRNA known from two studies of developing rice seeds (Zhu et al., 2008; Xue et al., 2009). The location of the miRNA match in the sequence is close to the 5′ end in all cases (120–200 bp). However, there is no significant homology among the three ncRNAS, which also vary in the number of predicted splice variants by a wide range (3, 7, and 20). Furthermore, the location of the miRNA near the 5′ end precludes any affect of AS on the presence or removal of the miRNA. It is possible that there are variant-specific effects on the stability or accessibility of the miRNA.

**Figure 6:**
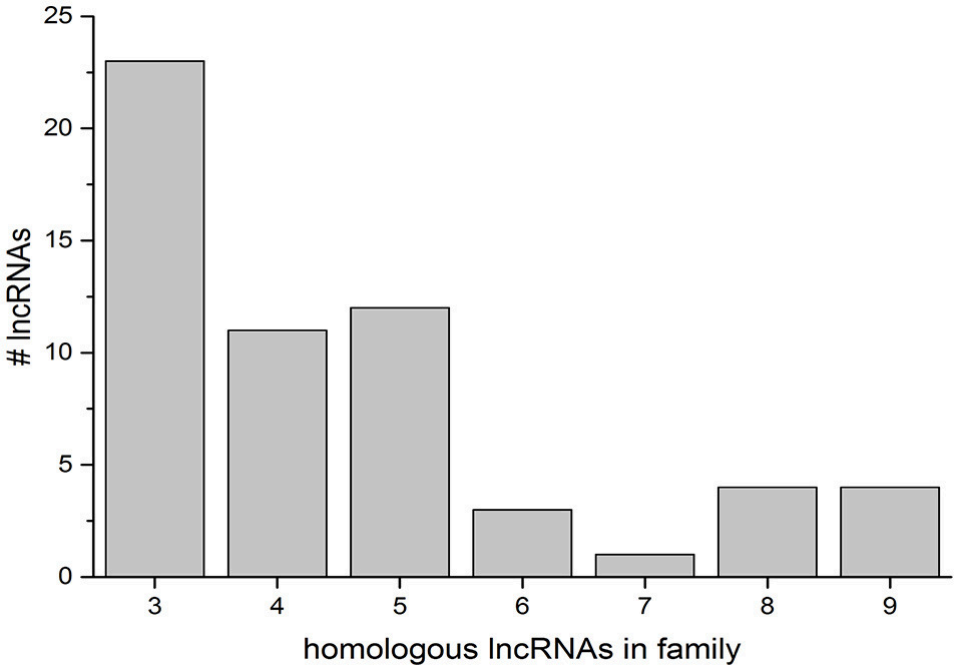
Groups of homologous lncRNAs in the NCBI rice genome. Gene families were defined as three or more lncRNAs sharing significant matches of 100 bp or more. The number of searched lncRNAs that fall into a given gene family size is indicated on the y-axis.

The discovery of conserved homologous regions in lncRNAs on multiple chromosomes was further studied with a computational analysis of the lncRNA population present in the NCBI genome annotation. All lncRNAs were sequestered from other RNA present in the annotation and used to search the ref_seq RNA database. Significant matches were then parsed into matches between lncRNAs, matches to coding RNAs, and matches in other plants. To determine the presence of lncRNA gene families, the output was limited to matches greater than 100 bp (Figure 6). The preponderance of gene families (with more than two sequence matches) is highest at low numbers, with 23 groups with three homologous lncRNAs. The most abundant families contain 3–5 members (46 families), with few containing 6 or more (12 families) (Figure 6, LOC IDs in Supplementary Data Sheet 3).

## Discussion

Alternative splicing is commonly quantified after RNA-seq experiments, but is rarely explored further due to challenges in validation of AS events in plants, as well as the relative lack of knowledge of the importance of AS in growth, development, or stress response in plants. Here we employed rMATs to assay publicly-available RNA-seq experiments, expecting a role for AS in early reproductive development of rice. However, there was instead a striking predominance of AS during seed development. This is consistent with data from rice and maize that indicate a role of AS in regulation of parent-specific epigenetic effects (e.g., in endosperm) (Luo et al., 2011) or control of splicing by methylation (Regulski et al., 2013). In fact, we noted large numbers of predicted AS events in datasets from reproductive cells and from a study of methylase *OsMet1-2* mutant compared to wild type seedlings (Figure 1A).

We corroborate previous observations that AS often involves components of the splicing machinery that are themselves subject to high levels of AS. Alternative splicing of pre-mRNA of *Arabidopsis* serine/arginine (SR) proteins has been known for more than a decade (Lazar and Goodman, 2000). Serine-arginine-rich proteins have been shown to be involved in regulation of alternative splicing in rice (Isshiki et al., 2006) and are themselves subject to extensive alternative splicing in *Arabidopsis* and other plants (Rauch et al., 2014; Palusa and Reddy, 2015). Analysis of AS in Arabidopsis pollen showed that in male gametes, the only significant difference in gene class of alternatively spliced transcripts were those of proteins involved in RNA processing (Loraine et al., 2013). More recently, it was shown that splicing-related genes undergo AS upon changes in ambient temperatures in plants (Verhage et al., 2017). Indeed, gene ontology analysis of AS targets predicted by rMATS revealed 5.5-fold enrichment in RNA splicing via spliceosome in reproductive cells, greater than ten-fold and seven-fold enrichment in two types of RNA splicing functions in *OsMet1-2* mutants and in developing seeds, respectively (Table 1). Interestingly, AS of Ser/Arg-rich splicing factor RSZ21 (LOC4330968) was shared by the three datasets (Figure 1B).

We used skipped exon events to assay AS predicted by rMATS. Because the program was designed to analyze mammalian genomes, in which skipped exons are the most prevalent AS event, retained introns are underrepresented in rMATS output (it is possible to make a specialized RI.gtf file to cover unannotated introns) (Guo et al., 2014). For this reason, the data presented for total splice events are likely underestimates, as retained introns are the predominant AS event in plants. SE events are simpler to assay, as PCR can be used to determine the presence of exon-inclusion and exon-skipping RNAs simultaneously in a single reaction, an approach used previously to determine un-annotated variants of SR genes in sorghum and maize, and multiple splice forms in Arabidopsis pollen (Rauch et al., 2014; Estrada et al., 2015). An example of a protein-coding gene, MORC7 (LOC4329338), undergoing complex exon skipping is shown in Figure 2. The largest variant predominates in milk seed, with skipping of one or more exons prevalent in embryo and endosperm of mature seed. In Arabidopsis, MORC family ATPases catalyze changes in chromatin structure, inducing gene silencing via RNA-directed DNA methylation, though MORC7 is also able to suppress genes in a methylation-independent manner (Moissiard et al., 2012; Harris et al., 2016).

rMATS also predicted alternative splicing of long non-coding RNAs during seed development. It has been shown that many lncRNAs are specifically expressed during rice flower and seed development (Zhang et al., 2014). We observed that many lncRNAs in the genome assembly are predicted to have multiple splice variants, to a much larger degree than predicted protein coding RNAs. In humans, single-exon, and multiple-exon lncRNAs are implicated in different functions (Chen et al., 2014). Using the rMATS data, we sought to elucidate the nature and extent of AS of rice seed lncRNAs.

LOC9270896 was predicted by rMATS to undergo both SE and RI events, and to produce 20 possible transcript variants, varying in length from 3,714 to 4,616 bp (Figure 3A). Using primers spanning three introns, it was possible to detect 5 AS transcripts. Intron processing was marked in embryo, which produced an unannotated variant lacking all introns (Figure 3B). This shorter transcript was the only variant detected in endosperm. In contrast, exon skipping was seen only in milk seed, with exon inclusion and an unannotated 5′ alternative splice site seen in embryo (Figure 3C). Thus, lncRNAs undergo extensive AS in rice seed, over and above the many variants predicted by the genome annotation. Furthermore, peptide-coding prediction suggests that LOC9270896 may be regulating peptide expression through selective intron and exon retention.

Another lncRNA with complex AS has only three predicted variants ranging in size from 1,080 to 1,406 bp (Figure 4A). LOC107279876 showed the opposite trend, with longer transcripts predominating in embryo (Figure 4B). More interesting, however, was the presence of sequence similarity to other lncRNAs in the genome (Figure 4C). After further analysis, it was found that the presence of lncRNA gene families was pervasive in the rice genome (discussed below). A broader survey of (simple) SE in eight additional lncRNAs demonstrated variability in the nature and extent of AS (Figure 5A). Relative to milk seed, embryo generally had more exon inclusion and endosperm more skipped exon events. All but one of the lncRNAs had more than one homologous lncRNAs in the genome (Figure 5B). In each case, we performed a search to determine if the predicted spliced regions of the lncRNAs were homologous to coding genes. The RI of lncRNA LOC9266258 matches a shaggy-related protein kinase (LOC4327108). Remarkably, the SE of lncRNA LOC9267988 and the RI of lncRNA LOC4331211 both matched an unknown protein (LOC9269363) (Figure 5B). The unknown protein is on chromosome 4, while the two lncRNAs are on chromosomes 1 and 2, respectively.

To elucidate the presence of gene families of lncRNAs in rice, we determined the presence or absence of homologs to each lncRNA in the genome by a blastn search against refseq_rna. Only highly significant hits with a minimum match length of 100 bp were considered. There were 10 lncRNAs in the genome that matched a single other lncRNA with these criteria, and 23 lncRNAs that matched two other lncRNAs in the genome. A “gene family” was considered to have at least three members. Small groups of 3–5 members were most common, although 12 groups with 6 or more members were observed (Figure 6). There was no correlation between the number of splice variants for a searched locus and the size of the gene families.

Using rMATS-generated predictions of AS in seeds and RT-PCR as a basis for the analysis means the RNAs in this study are by definition multi-exonic and polyadenylated. This means that ncRNAs lacking polyadenylation as well as RNAs with a single sequence variant are not present in the comparison of reproductive stages. In the case of the *COOLAIR* ncRNA, both proximal and distal polyadenation occurs. Disruption of a central component of the spliceosome (PRP8) revealed a specific effect on proximally polyadenylated COOLAIR transcripts (Marquardt et al., 2014). Overall, the polyadenation state and AS of *COOLAIR* are key regulatory elements in the control *FLC* expression (Wang et al., 2014). Similarly, the polyadenation state of the oxidative tolerant gene OXT6 controls the AS of the gene, the resulting proteins, and their functions (Li et al., 2017). Therefore, the lncRNAs in this study are a subset of the possible expression outcomes from these loci, which may express variants with other, functionally relevant, polyadenylationstates.

Many lncRNAs shared regions of significant homology to protein coding RNAs (5,581 significant matches). Because we were searching against RNA and not genomic sequence, it is possible that the homologous regions are involved in splicing competition or competition for other factors necessary for RNA processing. As mentioned above, in some cases regions with homology to coding RNAs can be found in exons and introns that are selectively spliced from lncRNAs. During Arabidopsis root development, lncRNAs affect AS of transcripts by competing with RNA-binding proteins and modulating the output of splice variants (Bardou et al., 2014). The widespread and conserved (sense) similarity between regions of the lncRNAs in this study and coding mRNA sequences in the genome annotation imply a similar function for the lncRNAs in this study.

Taken as a whole, these data demonstrate extensive AS during rice seed development that includes complex processing of lncRNAs. Using the same RNA-seq data, Wang et al. (2015) employed an elegant positional approach to infer lncRNA function by proximity to protein-coding genes. Their annotation contained 22,334 long intergenic non-coding RNAs, and the NCBI annotation we used contained 5,515 lncRNAs. While it has been shown in mouse that AS of a lncRNA, independent of overall sequence, can regulate a proximal (5 kb) coding transcript, our data suggest another “long distance” functional role for lncRNAs that is likely based on (conserved) sequence. The nature of gene families of lncRNAs that undergo AS during seed development deserves further study. Controlled mis-expression of lncRNA transcript variants would empower functional analysis of these enigmatic molecules.

## Author contributions

EK and EL performed molecular biology experiments, designed primers, developed RNA protocols; AG wrote scripts and performed bioinformatic analyses; MK designed the study, reviewed the results and oversaw the manuscript preparation.

### Conflict of interest statement

The authors declare that the research was conducted in the absence of any commercial or financial relationships that could be construed as a potential conflict of interest.

## References

[B1] Anderson S. N. Johnson C. S. Jones D. S. Conrad L. J. Gou X. Russell S. D.. (2013). Transcriptomes of isolated *Oryza sativa* gametes characterized by deep sequencing: evidence for distinct sex-dependent chromatin and epigenetic states before fertilization. J. Plant 76, 729–741. 10.1111/tpj.12336 24215296

[B2] Bardou F. Ariel F. Simpson C. G. Romero-Barrios N. Laporte P. Balzergue S.. (2014). Long noncoding RNA modulates alternative splicing regulators in Arabidopsis. Dev. Cell 30, 166–176. 10.1016/j.devcel.2014.06.017 25073154

[B3] Chen F. C. Pan C. L. Lin H. Y. (2014). Functional implications of RNA splicing for human long intergenic noncoding RNAs. Evol. Bioinform. Online 10, 219–228. 10.4137/EBO.S20772 25574121 PMC4264600

[B4] Copetti D. Zhang J. El Baidouri M. Gao D. Wang J. Barghini E.. (2015). RiTE database: a resource database for genus-wide rice genomics and evolutionary biology. BMC Genomics 16:538. 10.1186/s12864-015-1762-3 26194356 PMC4508813

[B5] Csorba T. Questa J. I. Sun Q. Dean C. (2014). Antisense COOLAIR mediates the coordinated switching of chromatin states at FLC during vernalization. Proc. Natl. Acad. Sci. U.S.A. 111, 16160–16165. 10.1073/pnas.1419030111 25349421 PMC4234544

[B6] Ding J. Lu Q. Ouyang Y. Mao H. Zhang P. Yao J.. (2012). A long noncoding RNA regulates photoperiod-sensitive male sterility, an essential component of hybrid rice. Proc. Natl. Acad. Sci. U.S.A. 109, 2654–2659. 10.1073/pnas.1121374109 22308482 PMC3289353

[B7] Estrada A. D. Freese N. H. Blakley I. C. Loraine A. E. (2015). Analysis of pollen-specific alternative splicing in *Arabidopsis thaliana* via semi-quantitative PCR. PeerJ 3:e919. 10.7717/peerj.919 25945312 PMC4419537

[B8] Gulledge A. A. Roberts A. D. Vora H. Patel K. Loraine A. E. (2012). Mining *Arabidopsis thaliana* RNA-seq data with integrated genome browser reveals stress-induced alternative splicing of the putative splicing regulator SR45a. Am. J. Bot. 99, 219–231. 10.3732/ajb.1100355 22291167

[B9] Guo R. Zheng L. Park J. W. Lv R. Chen H. Jiao F.. (2014). BS69/ZMYND11 reads and connects histone H3.3 lysine 36 trimethylation-decorated chromatin to regulated pre-mRNA processing. Mol. Cell 56, 298–310. 10.1016/j.molcel.2014.08.022 25263594 PMC4363072

[B10] Han R. Jian C. Lv J. Yan Y. Chi Q. Li Z.. (2014). Identification and characterization of microRNAs in the flag leaf and developing seed of wheat (*Triticum aestivum* L.). BMC Genomics 15:289. 10.1186/1471-2164-15-289 24734873 PMC4029127

[B11] Harris C. J. Husmann D. Liu W. Kasmi F. E. Wang H. Papikian A.. (2016). Arabidopsis AtMORC4 and AtMORC7 form nuclear bodies and repress a large number of protein-coding genes. PLoS Genet. 12:e1005998. 10.1371/journal.pgen.1005998 27171361 PMC4865129

[B12] Harrop T. W. Ud Din I. Gregis V. Osnato M. Jouannic S. Adam H.. (2016). Gene expression profiling of reproductive meristem types in early rice inflorescences by laser microdissection. Plant J. Cell Mol. Biol. 86, 75–88. 10.1111/tpj.13147 26932536

[B13] Isshiki M. Tsumoto A. Shimamoto K. (2006). The serine/arginine-rich protein family in rice plays important roles in constitutive and alternative splicing of pre-mRNA. Plant Cell 18, 146–158. 10.1105/tpc.105.037069 16339852 PMC1323490

[B14] Kalvari I. Argasinska J. Quinones-Olvera N. Nawrocki E. P. Rivas E. Eddy S. R. (2018) Rfam 13.0: shifting to a genome-centric resource for non-coding RNA families. Nucleic Acids Res. 46, D335–D342. 10.1093/nar/gkx1038 29112718 PMC5753348

[B15] Kang Y. J. Yang D. C. Kong L. Hou M. Meng Y. Q. Wei L.. (2017). CPC2: a fast and accurate coding potential calculator based on sequence intrinsic features. Nucleic Acids Res. 45. W12–W16. 10.1093/nar/gkx428 28521017 PMC5793834

[B16] Komiya R. Ohyanagi H. Niihama M. Watanabe T. Nakano M. Kurata N.. (2014). Rice germline-specific argonaute MEL1 protein binds to phasiRNAs generated from more than 700 lincRNAs. Plant J. Cell Mol. Biol. 78, 385–397. 10.1111/tpj.12483 24635777

[B17] Kozomara A. Griffiths-Jones S. (2014). miRBase: annotating high confidence microRNAs using deep sequencing data. Nucleic Acids Res. 42, D68–D73. 10.1093/nar/gkt1181 24275495 PMC3965103

[B18] Lazar G. Goodman H. M. (2000). The Arabidopsis splicing factor SR1 is regulated by alternative splicing. Plant Mol. Biol. 42, 571–581. 10.1023/A:1006394207479 10809003

[B19] Li Q. Q. Liu Z. Lu W. Liu M. (2017). Interplay between alternative splicing and alternative polyadenylation defines the expression outcome of the plant unique OXIDATIVE TOLERANT-6 Gene. Sci. Rep. 7:2052. 10.1038/s41598-017-02215-z 28515442 PMC5435732

[B20] Li S. Yamada M. Han X. Ohler U. Benfey P. N. (2016). High-resolution expression map of the Arabidopsis root reveals alternative splicing and lincRNA regulation. Dev. Cell 39, 508–522. 10.1016/j.devcel.2016.10.012 27840108 PMC5125536

[B21] Ling Y. Alshareef S. Butt H. Lozano-Juste J. Li L. Galal A. A.. (2017). Pre-mRNA splicing repression triggers abiotic stress signaling in plants. Plant J. Cell Mol. Biol. 89, 291–309. 10.1111/tpj.13383 27664942

[B22] Liu R. Loraine A. E. Dickerson J. A. (2014). Comparisons of computational methods for differential alternative splicing detection using RNA-seq in plant systems. BMC Bioinformatics 15:364. 10.1186/s12859-014-0364-4 25511303 PMC4271460

[B23] Liu Z. Qin J. Tian X. Xu S. Wang Y. Li H.. (2017). Global profiling of alternative splicing landscape responsive to drought, heat and their combination in wheat (*Triticum asetivum* L.). Plant Biotechnol. J. [Epub ahead of print]. 10.1111/pbi.12822 28834352 PMC5814593

[B24] Loraine A. E. McCormick S. Estrada A. Patel K. Qin P. (2013). RNA-Seq of arabidopsis pollen uncovers novel transcription and alternative splicing. Plant Phys. 162, 1092–1109. 10.1104/pp.112.211441 23590974 PMC3668042

[B25] Luo M. Taylor J. M. Spriggs A. Zhang H. Wu X. Russell S.. (2011). A genome-wide survey of imprinted genes in rice seeds reveals imprinting primarily occurs in the endosperm. PLoS Genet. 7:e1002125. 10.1371/journal.pgen.1002125 21731498 PMC3121744

[B26] Marquardt S. Raitskin O. Wu Z. Liu F. Sun Q. Dean C. (2014). Functional consequences of splicing of the antisense transcript COOLAIR on FLC transcription. Mol. Cell 54, 156–165. 10.1016/j.molcel.2014.03.026 24725596 PMC3988885

[B27] Mei W. Boatwright L. Feng G. Schnable J. C. Barbazuk W. B. (2017a). Evolutionarily conserved alternative splicing across monocots. Genetics 207, 465–480. 10.1534/genetics.117.300189 28839042 PMC5629316

[B28] Mei W. Liu S. Schnable J. C. Yeh C. T. Springer N. M. Schnable P. S.. (2017b). A comprehensive analysis of alternative splicing in paleopolyploid maize. Front. Plant Sci. 8:694. 10.3389/fpls.2017.00694 28539927 PMC5423905

[B29] Mi H. Huang X. Muruganujan A. Tang H. Mills C. Kang D.. (2017). PANTHER version 11: expanded annotation data from Gene Ontology and Reactome pathways, and data analysis tool enhancements. Nucleic Acids Res. 45, D183–D189. 10.1093/nar/gkw1138 27899595 PMC5210595

[B30] Moissiard G. Cokus S. J. Cary J. Feng S. Billi A. C. Stroud H.. (2012). MORC family ATPases required for heterochromatin condensation and gene silencing. Science 336, 1448–1451. 10.1126/science.1221472 22555433 PMC3376212

[B31] Notredame C. Higgins D. G. Heringa J. (2000). T-coffee: a novel method for fast and accurate multiple sequence alignment. J. Mol. Biol. 302, 205–217. 10.1006/jmbi.2000.4042 10964570

[B32] Palusa S. G. Reddy A. S. (2015). Differential recruitment of splice variants from SR pre-mRNAs to polysomes during development and in response to stresses. Plant Cell Physiol. 56, 421–427. 10.1093/pcp/pcv010 25637375

[B33] Park K. Kim M. Y. Vickers M. Park J. S. Hyun Y. Okamoto T.. (2016). DNA demethylation is initiated in the central cells of Arabidopsis and rice. Proc. Natl. Acad. Sci. U.S.A. 113, 15138–15143. 10.1073/pnas.1619047114 27956642 PMC5206524

[B34] Qüesta J. I. Song J. Geraldo N. An H. Dean C. (2016). Arabidopsis transcriptional repressor VAL1 triggers Polycomb silencing at FLC during vernalization. Science 353, 485–488. 10.1126/science.aaf7354 27471304

[B35] Rauch H. B. Patrick T. L. Klusman K. M. Battistuzzi F. U. Mei W. Brendel V. P.. (2014). Discovery and expression analysis of alternative splicing events conserved among plant SR proteins. Mol. Biol. Evol. 31, 605–613. 10.1093/molbev/mst238 24356560

[B36] Reddy A. S. (2007). Alternative splicing of pre-messenger RNAs in plants in the genomic era. Annu. Rev. Plant Biol. 58, 267–294. 10.1146/annurev.arplant.58.032806.103754 17222076

[B37] Reddy A. S. Rogers M. F. Richardson D. N. Hamilton M. Ben-Hur A. (2012). Deciphering the plant splicing code: experimental and computational approaches for predicting alternative splicing and splicing regulatory elements. Front. Plant Sci. 3:18. 10.3389/fpls.2012.00018 22645572 PMC3355732

[B38] Regulski M. Lu Z. Kendall J. Donoghue M. T. Reinders J. Llaca V.. (2013). The maize methylome influences mRNA splice sites and reveals widespread paramutation-like switches guided by small RNA. Genome Res. 23, 1651–1662. 10.1101/gr.153510.112 23739895 PMC3787262

[B39] Rodrigues J. A. Ruan R. Nishimura T. Sharma M. K. Sharma R. Ronald P. C.. (2013). Imprinted expression of genes and small RNA is associated with localized hypomethylation of the maternal genome in rice endosperm. Proc. Natl. Acad. Sci. U.S.A. 110, 7934–7939. 10.1073/pnas.1306164110 23613580 PMC3651473

[B40] Shen S. Park J. W. Lu Z. X. Lin L. Henry M. D. Wu Y. N.. (2014). rMATS: robust and flexible detection of differential alternative splicing from replicate RNA-Seq data. Proc. Natl. Acad. Sci. U.S.A. 111, E5593–E5601. 10.1073/pnas.1419161111 25480548 PMC4280593

[B41] Simpson C. G. Fuller J. Calixto C. P. McNicol J. Booth C. Brown J. W.. (2016). Monitoring alternative splicing changes in Arabidopsis circadian clock genes. Methods Mol. Biol. 1398, 119–132. 10.1007/978-1-4939-3356-3_11 26867620

[B42] Tokheim C. Park J. W. Xing Y. (2014). PrimerSeq: design and visualization of RT-PCR primers for alternative splicing using RNA-seq data. Genomics Proteomics Bioinformatics 12, 105–109. 10.1016/j.gpb.2014.04.001 24747190 PMC4411361

[B43] Ulitsky I. (2016). Evolution to the rescue: using comparative genomics to understand long non-coding RNAs. Nat. Rev. Genet. 17, 601–614. 10.1038/nrg.2016.85 27573374

[B44] Verhage L. Severing E. I. Bucher J. Lammers M. Busscher-Lange J. Bonnema G.. (2017). Splicing-related genes are alternatively spliced upon changes in ambient temperatures in plants. PLoS ONE 12:e0172950. 10.1371/journal.pone.0172950 28257507 PMC5336241

[B45] Wang G. Wang G. Zhang X. Wang F. Song R. (2012). Isolation of high quality RNA from cereal seeds containing high levels of starch. Phytochem. Anal. 23, 159–163. 10.1002/pca.1337 21739496

[B46] Wang H. V. Chekanova J. A. (2017). Long noncoding RNAs in plants. Adv. Exp. Med. Biol. 1008, 133–154. 10.1007/978-981-10-5203-3_5 28815539 PMC6689229

[B47] Wang H. Niu Q. W. Wu H. W. Liu J. Ye J. Yu N.. (2015). Analysis of non-coding transcriptome in rice and maize uncovers roles of conserved lncRNAs associated with agriculture traits. Plant J. Cell Mol. Biol. 84, 404–416. 10.1111/tpj.13018 26387578

[B48] Wang X. Hu L. Wang X. Li N. Xu C. Gong L.. (2016). DNA methylation affects gene alternative splicing in plants: an example from rice. Mol. Plant 9, 305–307. 10.1016/j.molp.2015.09.016 26455460

[B49] Wang Z. W. Wu Z. Raitskin O. Sun Q. Dean C. (2014). Antisense-mediated FLC transcriptional repression requires the P-TEFb transcription elongation factor. Proc. Natl. Acad. Sci. U.S.A. 111, 7468–7473. 10.1073/pnas.1406635111 24799695 PMC4034230

[B50] Wei H. Lou Q. Xu K. Yan M. Xia H. Ma X.. (2017). Alternative splicing complexity contributes to genetic improvement of drought resistance in the rice maintainer HuHan2B. Sci. Rep. 7:11686. 10.1038/s41598-017-12020-3 28916800 PMC5601427

[B51] Xin R. Zhu L. Salomé P. A. Mancini E. Marshall C. M. Harmon F. G.. (2017). SPF45-related splicing factor for phytochrome signaling promotes photomorphogenesis by regulating pre-mRNA splicing in Arabidopsis. Proc. Natl. Acad. Sci. U.S.A. 114, E7018–E7027. 10.1073/pnas.1706379114 28760995 PMC5565451

[B52] Xue L. J. Zhang J. J. Xue H. W. (2009). Characterization and expression profiles of miRNAs in rice seeds. Nucleic Acids Res. 37, 916–930. 10.1093/nar/gkn998 19103661 PMC2647296

[B53] Zemach A. Kim M. Y. Silva P. Rodrigues J. A. Dotson B. Brooks M. D.. (2010). Local DNA hypomethylation activates genes in rice endosperm. Proc. Natl. Acad. Sci. U.S.A. 107, 18729–18734. 10.1073/pnas.1009695107 20937895 PMC2972920

[B54] Zhang Y. C. Liao J. Y. Li Z. Y. Yu Y. Zhang J. P. Li Q. F.. (2014). Genome-wide screening and functional analysis identify a large number of long noncoding RNAs involved in the sexual reproduction of rice. Genome Biol. 15:512. 10.1186/s13059-014-0512-1 25517485 PMC4253996

[B55] Zhu Q.-H. Spriggs A. Matthew L. Fan L. Kennedy G. Gubler F.. (2008). A diverse set of microRNAs and microRNA-like small RNAs in developing rice grains. Genome Res. 18, 1456–1146. 10.1101/gr.075572.107 18687877 PMC2527712

